# Prenatal methamphetamine exposure causes dysfunction in glucose metabolism and low birthweight

**DOI:** 10.3389/fendo.2022.1023984

**Published:** 2022-10-24

**Authors:** Miyuki Doi, Nanako Nakama, Takuya Sumi, Noriyoshi Usui, Shoichi Shimada

**Affiliations:** ^1^ Department of Neuroscience and Cell Biology, Graduate School of Medicine, Osaka University, Suita, Japan; ^2^ Addiction Research Unit, Osaka Psychiatric Research Center, Osaka Psychiatric Medical Center, Osaka, Japan; ^3^ Department of Cell Biology, Graduate School of Medicine, Osaka University, Osaka, Japan; ^4^ United Graduate School of Child Development, Osaka University, Suita, Japan; ^5^ Global Center for Medical Engineering and Informatics, Osaka University, Suita, Japan

**Keywords:** methamphetamine, addiction, drug abuse, low birthweight (LBW), metabolism, placenta, embryonic development

## Abstract

Methamphetamine (METH) is a psychostimulant drug that induces addiction. Previous epidemiological studies have demonstrated that maternal METH abuse during pregnancy causes low birthweight (LBW) in the offspring. As a source of essential nutrients, in particular glucose, the placenta plays a key role in fetal development. LBW leads to health problems such as obesity, diabetes, and neurodevelopmental disorders (NDDs). However, the detailed mechanism underlying offspring’s LBW and health hazards caused by METH are not fully understood. Therefore, we investigated the effects of prenatal METH exposure on LBW and fetal-placental relationship by focusing on metabolism. We found dysfunction of insulin production in the pancreas of fetuses exposed to METH. We also found a reduction of the glycogen cells (GCs) storing glycogens in the junctional zone of placenta, all of which suggest abnormal glucose metabolism affects the fetal development. These results suggest that dysfunction in fetal glucose metabolism may cause LBW and future health hazards. Our findings provide novel insights into the cause of LBW *via* the fetal-placental crosstalk.

## Introduction

Epidemiological studies have demonstrated that disturbances in the prenatal environment, such as drug exposure and undernutrition during pregnancy, influence the offspring’s health at later stages (1). This theory is called the developmental origins of health and disease (DOHaD) theory (1–3). For example, in the US, there are approximately 2.4 million cocaine users (4), and cocaine abuse among pregnant women causes severe health problems in offspring including fetal death, malformation, preterm birth, LBW, and poisoning symptom after birth (5).

In general, methamphetamine (METH) not only enhances dopamine signaling in the reward system (6), but also affects noradrenaline and serotonin signaling (6). Disturbances of these signaling in the central nervous system (CNS) and/or in other organs by METH, eventually resulting in mental problems such as arousal, anxiety, and delusions (6). In particular, METH acts directly on the fetus, because it can cross the blood-placental barrier. METH abuse during pregnancy also causes severe health problems in offspring such as malformation, preterm birth, and LBW (7, 8). However, the detailed mechanisms underlying the influences of prenatal METH exposure on birth and offspring’s health are not fully understood.

Placenta is the most important organ for fetal development, playing an essential role in providing oxygen, vitamins, lipids, minerals, amino acids, glucose, and other nutrients to the fetus (9). Placenta also functions as a barrier, and protects the fetus from toxic substances (10). On the other hand, placental dysfunction is caused by inflammation (11), stress (12), and steroid imbalance (13), which leads to severe problems in fetal development. For example, maternal immune activation (MIA) induced by lipopolysaccharide leads to placental inflammation and miscarriage (11).

Mouse placenta consists of three layers namely: decidua zone, junctional zone, and labyrinth zone, each of which has different functions. The decidua zone is composed of stromal cells and acts as a physical barrier (10). Junctional zone is composed of spongiotrophoblasts and GCs, where synthases and stores glycogen as nutrients for the fetus. GCs supply nutrition to the fetus in the form of glucose (10, 14). The glucose transporters (GLUTs) play a crucial role in placental glucose uptake and supply in a facilitated diffusion manner. The expression level of GLUT genes in the mouse placenta changes over the course of pregnancy. Importantly, placental glycogen synthesis is regulated by insulin secreted from the fetal pancreas (15). In addition, labyrinth zone functions as blood-placental barrier, and has maternal vascular sinusoids and fetal vessels where exchange of nutrients and oxygen takes place (16). However, the molecular actions of METH on placental function are not fully understood.

In this study, we therefore investigated the effect of maternal METH abuse during pregnancy on the placenta as well as the effects of prenatal METH exposure on fetal development particularly LBW. We evaluated fetal development, insulin production from fetal pancreas and the placental functions by using a prenatal METH exposure mouse model.

## Methods

### Mice

All procedures were performed in accordance with the ARRIVE guidelines. The animal study was reviewed and approved by the Animal Research Committee of Osaka University (approval number #27-010). Five pregnant female C57BL/6J mice (Japan SLC Inc., Shizuoka, Japan) in each group were used in this study. For embryo staging, the day of vaginal plug detection was considered embryonic day (E) 0.5. Mice were housed in groups of two animals per cage (143 mm × 293 mm × 148 mm) in the barrier facilities of Osaka University under a 12 h light–dark cycle and given free access to water and food. An experimenter blinded to the group setting performed all the tests.

### METH administration

Ten mg/kg METH (#871151; Sumitomo Pharma, Osaka, Japan) dissolved in saline (#3311401A2026; Otsuka Pharmaceutical Co., Ltd., Tokyo, Japan) was injected intraperitoneally into the mice on E12.5, E14.5, and E16.5. The blood METH concentration in abusers is 0.3 mg/L (17), 10 mg/kg METH is daily accumulated dose used by METH abusers (18). Thus, the dose of METH is approximately equivalent to human METH abusers. Mice in the control group were injected with saline instead of METH dissolved in saline. The mice were analyzed on E18.5.

### Immunohistochemistry

The placentas and fetal pancreases were randomly selected from the litters in each pregnant mother mouse. The placentas and fetal pancreases of the mice were fixed in 4% paraformaldehyde (PFA) in phosphate buffered saline (PBS) overnight at 4°C and then cryoprotected in 30% sucrose in PBS for 48-96 h at 4°C. Samples were embedded in Tissue-Tek O.C.T. Compound (#4583, Sakura Finetek Japan Co.,Ltd., Osaka, Japan) and then sliced into 20 μm sections using a cryostat. Sections were mounted on Matsumami Adhesive Silan (MAS)-coated Glass Slides (#MAS-01; Matsunami Glass Ind.,Ltd., Osaka, Japan) and incubated with guinea pig polyclonal anti-insulin (Envision FLEX-Insulin) (1:2, #IR002, Agilent Technologies, Santa Clara, CA, USA) and anti-GYS1 (1:50, #10566-1-AP, Proteintech, Rosemont, IL, USA) primary antibodies. For fluorescence immunostaining, a species-specific antibody conjugated to Alexa Fluor 488 (1:2,000; Invitrogen, Carlsbad, CA, USA) was applied, and the cover glass was mounted with Fluoromount/Plus (#K048; Diagnostic BioSystems, Pleasanton, CA, USA). The nuclei were stained using 4,6-Diamidino-2-phenylindole Dihydrochloride (DAPI) (#11034-56; Nacalai Tesque, Kyoto, Japan). The stained sections were imaged using an all-in-one fluorescence microscope (BZ-X700; KEYENCE Corporation, Osaka, Japan). The intensity of immunopositive cells was quantified using KEYENCE analysis software with the Hybrid Cell Count Application (KEYENCE Corporation). The placental area was quantified manually using the ImageJ software.

### Hematoxylin-Eosin staining

HE staining was performed as described previously (11). The mouse placentas were fixed in 4% PFA overnight at 4°C and then embedded in paraffin. Paraffin sections (7 μm thick) of the central region of the placenta were deparaffinized and stained with Hematoxylin (#131-09665; FUJIFILM Wako Pure Chemical Corporation, Osaka, Japan) and 1% Eosin Y Solutions (#051-06515; FUJIFILM Wako Pure Chemical Corporation). The sections were mounted with Permount Mounting Medium (#SP15-100; Fisher Scientific, Pittsburgh, PA, USA) after dehydration. Stained sections were imaged using an all-in-one fluorescence microscope (BZ-X700; KEYENCE Corporation).

### Periodic Acid Schiff (PAS) staining

Mouse placentas were fixed in 4% PFA overnight 4°C and then embedded in paraffin. Paraffin sections (7μm thick) of the central region of the placenta were deparaffinized and treated with 0.5% Periodic Acid Solution (#164-19705; FUJIFILM Wako Pure Chemical Corporation), Schiff’s Reagent (#191-08441; FUJIFILM Wako Pure Chemical Corporation), and Sulfurous Acid Solution (#196-11005; FUJIFILM Wako Pure Chemical Corporation). The sections were then stained with Hematoxylin Solution (#131-09665; FUJIFILM Wako Pure Chemical Corporation). The sections were mounted with Permount Mounting Medium (#SP15-100; Fisher Scientific) after dehydration. The stained sections were imaged using an all-in-one fluorescence microscope (BZ-X700; KEYENCE Corporation).

### Quantitative real-time PCR

qPCR was performed as described previously (11, 19). Total RNA was extracted from the placenta using the miRNeasy Mini Kit (#217004; Qiagen, Hilden, Germany), according to the manufacturer’s instructions. Single-stranded cDNA was prepared using DNaseI, Amplification Grade (#18068015; Thermo Fisher Scientific, Waltham, MA), and SuperScript III First-Strand Synthesis Super Mix (#18080400; Thermo Fisher Scientific), and amplified by PCR according to the manufacturer’s instructions. qRT-PCR was performed using PowerUp SYBR Green Master Mix (#A25742; Thermo Fisher Scientific) and QuantStudio 7 Flex Real-Time PCR System (Thermo Fisher Scientific). Each biological sample had four technical replicates for qPCR, and the number of biological replicates for each experiment is indicated in the figure legend. As a reference for normalization, *18S* rRNA was used. Data were analyzed by the ΔΔCq method using QuantStudio 7 Flex Real-Time PCR System software (Thermo Fisher Scientific). The following primers were used: *18S rRNA*, F-5′-GAGGGAGCCTGAGAAACGG-3′, and R-5′-GTCGGGAGTGGGTAATTTGC-3′; *Prl7c1*, F-5’-GCTGCTGTCTTTGACTCATCC-3’, and R-5’-CAACAACATTGGGAGCATTG-3’. The cycling conditions were 50°C for 2 min and 95°C for 2 min, followed by 40 cycles at 95°C for 1 s and 60°C for 30 s, as per the manufacturer’s instructions.

### Statistical analysis

All data are presented as means of independent biological experiments ± standard error of the mean (SEM). Statistical analysis (unpaired *t*-test) was performed using the Prism 9. Asterisks indicate *P*-values (***P < 0.001, **P < 0.01, *P < 0.05). Statistical significance was set at *P* < 0.05.

## Results

### Prenatal METH exposure leads to LBW and insulin production defect from fetal pancreatic cells

**Figure 1:**
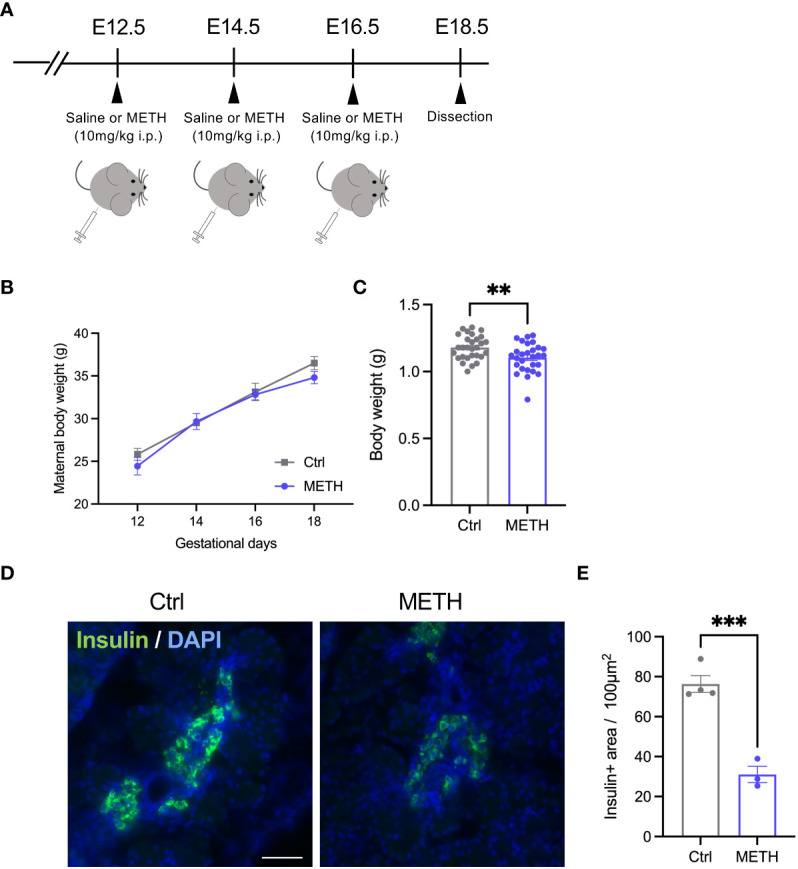
Prenatal METH exposure resulted in LBW and insulin production defect in the fetal pancreatic cells. **(A)** Experimental time course of prenatal METH exposure. METH or saline was administered intraperitoneally at E12.5, E14.5, and E16.5. At E18.5, pregnant mice were dissected. **(B)** No change in maternal body weight by METH administration. **(C)** Quantification of the body weight of offspring at E18.5. The body weight was significantly reduced in METH-exposed fetus. **(D)** Representative images of Insulin positive (+) pancreatic β cells. **(E)** Quantification of the Insulin + area. Insulin+ area was significantly decreased in METH-exposed fetal pancreas. Scale bar: 500 μm. Data are represented as means ( ± SEM). ****P < 0.0001, **P < 0.01, Two-way ANOVA with a Tukey’s multiple comparison test, unpaired *t*-test, n = 4/condition for maternal body weight, n = 28-29/condition for body weight, n = 3-4/condition for Insulin+ area.

To investigate the effects of prenatal METH exposure on fetal development, we injected 10 mg/kg METH into pregnant mice intraperitoneally at E12.5, E14.5, and E16.5, and sacrificed the fetus and placenta just before birth (E18.5) (**Figure 1A**). During these terms, no changes in the maternal body weights were observed (Ctrl: day12: 25.83 ± 0.90, day14: 29.50 ± 0.47, day16: 33.12 ± 1.22, day18: 36.50 ± 0.79, METH: day12: 24.43 ± 1.02, day14: 29.67 ± 0.93, day16: 32.80 ± 0.61, day18: 34.80 ± 0.70, *P*=0.16) (**Figure 1B**). We found a significant reduction in fetal weight (Ctrl: 1.18 ± 0.017, METH: 1.1 ± 0.02, *P*=0.0059), indicating that LBW was induced by prenatal METH exposure (**Figure 1C**). Furthermore, to investigate the effects of METH on fetal metabolism, we focused on insulin production in the fetal-placental crosstalk, and found that prenatal METH exposure induced defects in insulin production from the fetal pancreas (Ctrl: 76.36 ± 4.20, METH: 31.06 ± 4.07, *P*=0.0007) (**Figures 1D, E** **)**. These results indicate that insulin-related metabolism is abnormal in METH-exposed fetuses.

### Prenatal METH exposure decreases placental glycogen positive junctional zone

**Figure 2:**
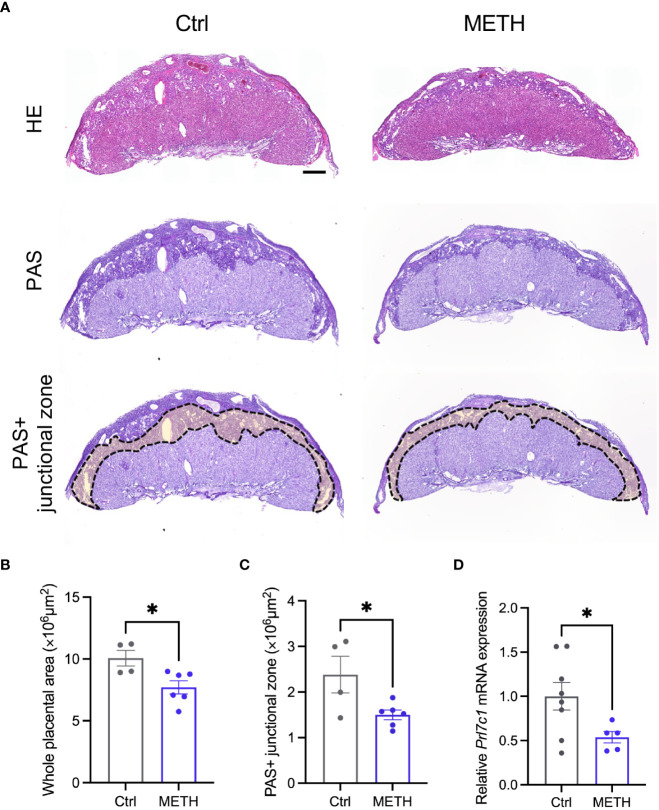
Prenatal METH exposure reduced the junctional zone area and glycogen in the placenta. **(A)** Representative images of HE staining (upper images), PAS staining (bottom images) and PAS+ junctional zone of placentas at E18.5. **(B)** Quantification of placental area. The whole placental area was significantly decreased by METH exposure. **(C)** Quantification of PAS+ junctional zone of the placentas. PAS+ glycogen cells (GCs) in the junctional zone were significantly reduced in METH-exposed placenta. **(D)** Quantification of *Prl7c1* mRNA expression. The *Prl7c1* expression was significantly reduced in METH-exposed placenta. Scale bar: 500 μm. Data are presented as means ( ± SEM). *P < 0.05, unpaired *t*-test, n = 4-6/condition for whole placental area, n = 4-6/condition for PAS+ junctional zone, and n = 5-8/condition for *Prl7c1* expression.

To investigate the abnormalities in insulin-related metabolism caused by prenatal METH exposure, we focused on the placenta supplying glucose to the fetus. We evaluated the placental morphologies, and found that the placental area was significantly decreased by METH exposure (**Figures 2A, B** **)**. PAS staining was performed to visualize GCs in the junctional zone (Ctrl: 10.06 ± 0.636, METH: 7.70 ± 0.534, *P*=0.0224) (**Figure 2A**). The PAS (glycogen) positive (+) junctional zone area was significantly decreased due to prenatal METH exposure (Ctrl: 2.38 ± 0.404, METH: 1.49 ± 0.106, *P*=0.0339) (**Figure 2C** **)**. We also evaluated the mRNA expression of *Prl7c1*, a marker gene of GCs. The mRNA expression of *Prl7c1* in METH-exposed placentas was found to be significantly reduced in comparison to that of the control placentas (Ctrl: 1.0 ± 0.156, METH: 0.54 ± 0.066, *P*=0.0471) (**Figure 2D**). Furthermore, we analyzed the expression of glycogen synthase 1 (GYS1) in the placenta to investigate whether prenatal METH exposure functionally altered glucose metabolism, and found the reduction of expression level of GYS1 in the METH-exposed placenta (Ctrl: 25.0 ± 6.557, METH: 6.75 ± 1.887, *P*=0.0274) (**Supplementary Figure 1**). Collectively, these results demonstrate that prenatal METH exposure caused loss of GCs in the junctional zone, suggesting the dysfunction in fetal glucose metabolism may result in LBW.

## Discussion

Numerous studies have reported that maternal METH abuse during pregnancy leads to LBW in offspring (7, 8). Furthermore, LBW induces metabolic dysfunctions such as obesity and diabetes (20). In this study, we showed that prenatal METH exposure resulted in fetal weight loss and insulin production defect in the pancreas of the fetus (**Figure 1**). We also found a decrease in the junctional zone containing GCs in the placenta after prenatal METH exposure (**Figure 2**). To the best of our knowledge, this study first demonstrates that prenatal METH exposure results in LBW with placental dysfunction involved in glucose metabolism.

Generally, it is known that taking METH causes the body weight loss. There are two major mechanisms in the body weight loss by METH intake. Firstly, reinforce of metabolic function *via* sympathetic nerve excitation. The other one is suppression of feeding center in hypothalamus. It is possible to reduce the maternal body weight, if fetal weight loss is attributed to such actions of METH. However, in this study, we did not observe maternal body weight loss by METH exposure (**Figure 1B**). Fetal nutrition during fetal period comes from the mother and placenta. Thus, it is suggested that LBW in offspring is triggered by placental dysfunction, not from the mother. In fact, prenatal METH exposure causes impaired placental blood flow *via* inhibition of placental monoamine transporters, resulting in impaired oxygen and nutrient supply and consequently placental dysfunction in human (21).

In the CNS, METH has various pharmacological actions, such as competitive inhibitions of monoamine transporters, acceleration of dopamine release from synaptic vesicles, and inhibition of monoamine oxidase. Outside of the CNS, pancreatic β cells express serotonin and dopamine receptors and both transporters regulating insulin secretion (22, 23). For example, dopamine D3 receptor activation suppresses insulin secretion by increased level of intracellular Ca^2+^ in the pancreatic β cells (24). Dopamine signaling also regulates insulin secretion through both adrenergic and dopamine receptors in the pancreatic β cells (25). In contrast, it is suggested that high concentration of extracellular serotonin inhibits insulin secretion *via* serotonin receptor 1a (HTR1A) (26). Previous study shows that the prenatal METH exposure does not induce the significant reduction of glucagon in offspring (27). Thus, it is thought that the reduction of insulin production is not caused by dysfunction of pancreatic β cells in this study. From these previous studies, it is possible that fetal insulin secretion regulated by serotonin and/or dopamine signaling in the pancreatic β cells is impaired by prenatal METH exposure.

We found that decreases of GCs in the junctional zone was caused by maternal METH exposure. Interestingly, regarding insulin production and/or secretion, response for METH administration is different fetus and adult. METH pass thorough blood-placental barrier (28), thus, it may affect fetal systemic insulin level directly. It is likely that systemic METH in the fetus increases serotonin and dopamine levels in pancreas and leads suppression of insulin secretion. Previous study reported that fetal blood concentration of METH is higher than that of pregnant mother (28). In addition, insulin can not pass through blood-placental barrier (29), but it is reported that insulin injection into the fetus increases placental glycogen store (30). In fact, the glucose in junctional zone is provided from the mother *via* facilitated diffusion from maternal circulation (10). Fetal systemic insulin level may reflects into the insulin level in the junctional zone because junctional zone is categorized in fetal tissue (10). Placenta is only the source of nutrients supplying to the fetus. Junctional zone containing glycogen is supplying glucose, which is essential for growth to the fetus. Previous study demonstrate that insulin secreted from the fetal pancreas promotes glycogen synthesis in the GCs of placenta (31). Thus, dysfunction of insulin secretion from the fetal pancreas caused by METH exposure may lead suppression of glycogen synthesis in the junctional zone, and eventually resulting in the LBW by the reduction of glucose supply.

LBW and preterm birth impair normal brain development and cause NDDs such as autism spectrum disorder (ASD) and attention deficit hyperactivity disorder (ADHD) (1). In this study, we demonstrated that prenatal METH exposure led to LBW. However, there are various other causes of LBW, such as maternal stress, undernutrition, and MIA. For example, MIA, a known risk factor for ASD, also causes LBW and placental inflammation (11, 32). MIA induces IL-17a expression from T-helper 17 cells in pregnant mothers. Maternal IL-17a reaches the fetal brain through the placenta during pregnancy and causes defects in the cortical laminar structure (32). Therefore, in future studies, the influences of LBW on the brain development and behaviors of offspring should be investigated in terms of association between LBW induced by prenatal METH exposure and NDDs.

We acknowledge the limitations of this study as follows; (1) we could not examine glucose changes and plasma insulin levels in METH exposure maternal blood, (2) fetal monoamines were not measure in METH-exposed placenta, (3) since focused on the mechanism of LBW, the effects of prenatal METH exposure on postnatal offspring could not be analyzed (from a DOHaD perspective), (4) possibility whether METH toxicity affected other organs. These questions are necessary to understand the effects of prenatal METH exposure and will be investigate as future studies.

In conclusion, our study suggests that the psychostimulant drug METH induces not only psychological dependence but also disturbances in the endocrine system, particularly glucose metabolism in the prenatal period. Previous study has reported that birthweight was reduced in children with prenatal METH expose compared with non-exposed children (8). Our study provides novel insights into the mechanism underlying LBW caused by prenatal METH exposure, which may contribute to the understanding of the DOHaD theory.

## Data availability statement

The original contributions presented in the study are included in the article/**Supplementary Material**. Further inquiries can be directed to the corresponding author.

## Author contributions

MD: Validation, Investigation, Writing - Original Draft, Writing - Review & Editing, Visualization, Funding acquisition. NN: Validation, Investigation. TS: Investigation. NU: Conceptualization, Methodology, Validation, Investigation, Writing – Original Draft, Writing – Review & Editing, Project administration, Funding acquisition. SS: Writing – Review & Editing, Supervision, Funding acquisition. All authors contributed to the article and approved the submitted version.

## Funding

This work was supported by Japan Society for the Promotion of Science (JSPS) Grant-in-Aid for Scientific Research (C) (20K06872) to NU. JSPS Grant-in-Aid for Challenging Research (20K21654) to NU and SS. Osaka Medical Research Foundation for Intractable Diseases to MD and NU, NN is supported by the Osaka University Medical Doctor Scientist Training Program.

## Acknowledgments

We thank the CentMeRE and CoMIT Omics Center, Graduate School of Medicine, Osaka University, for their support.

## Conflict of interest

The authors declare that the research was conducted in the absence of any commercial or financial relationships that could be construed as a potential conflict of interest.

## Publisher’s note

All claims expressed in this article are solely those of the authors and do not necessarily represent those of their affiliated organizations, or those of the publisher, the editors and the reviewers. Any product that may be evaluated in this article, or claim that may be made by its manufacturer, is not guaranteed or endorsed by the publisher.

## Supplementary material

Supplementary Figure 1 Glycogen synthase 1 (GYS1) expression was reduced in METH-exposed placenta. Representative images of GYS1 positive (+) regions in the placentas at E18.5 and its quantification in the placentas. The expression levels of GYS1 in the placenta was significantly reduced in METH-exposed placenta. Data are presented as means ( ± SEM). **P* < 0.05, unpaired *t*-test, n = 3-4/condition.
