# Supplementary figures and images for: Prenatal methamphetamine exposure causes dysfunction in glucose metabolism and low birthweight
Source: Front Endocrinol (Lausanne). 2022 Oct 24;13:1023984. doi: 10.3389/fendo.2022.1023984 (PMC9637823; doi:10.3389/fendo.2022.1023984)

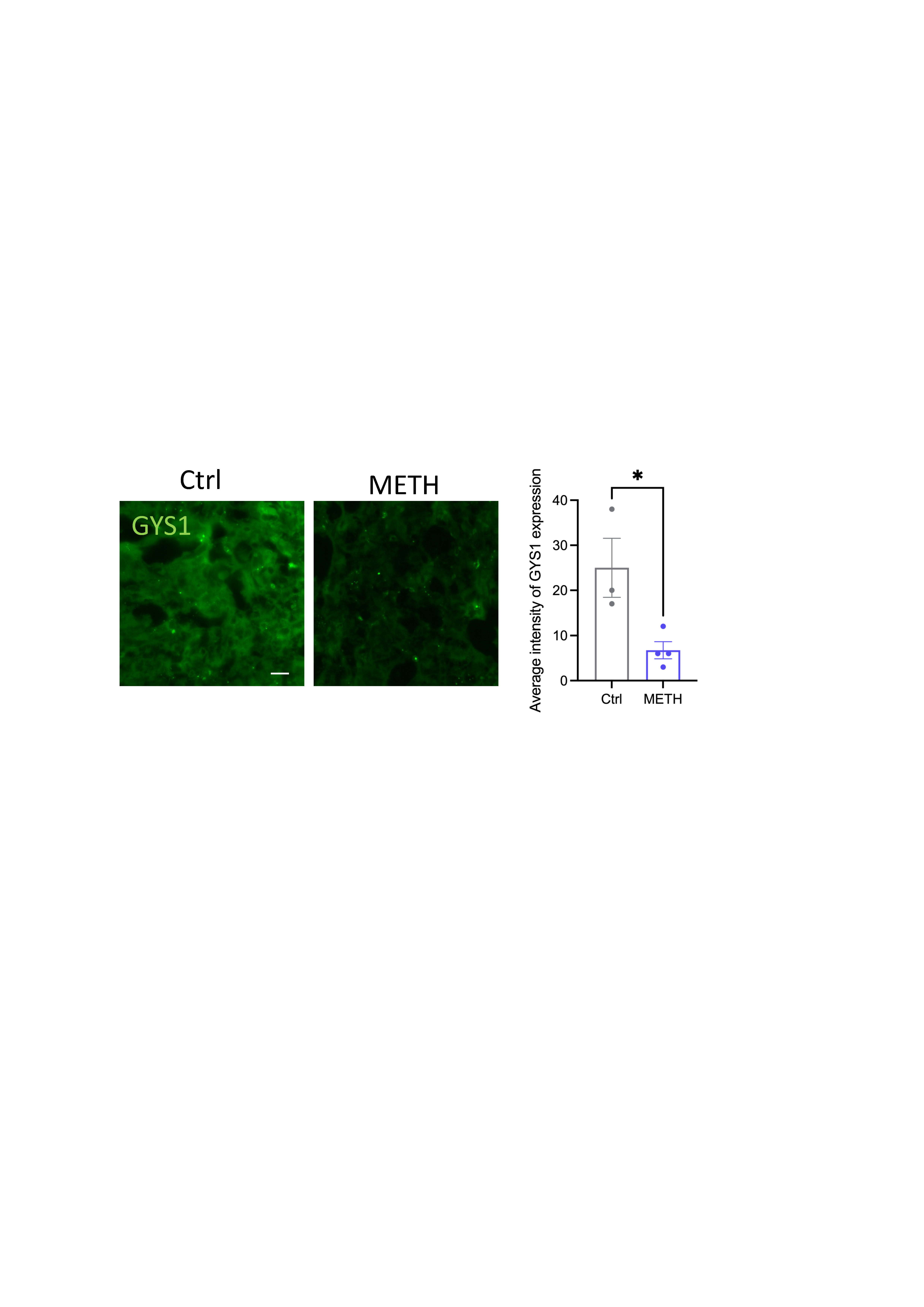

Supplement: Supplementary Figure 1 — Glycogen synthase 1 (GYS1) expression was reduced in METH-exposed placenta. Representative images of GYS1 positive (+) regions in the placentas at E18.5 and its quantification in the placentas. The expression levels of GYS1 in the placenta was significantly reduced in METH-exposed placenta. Data are presented as means ( ± SEM). *P < 0.05, unpaired t-test, n = 3-4/condition. [file Image_1.tiff]
